# Clutter costs in head-mounted displays: a study examining trade-offs between overlay and adjacent presentation of information

**DOI:** 10.1186/s41235-025-00650-5

**Published:** 2025-08-05

**Authors:** Amelia C. Warden, Christopher D. Wickens, Daniel Rehberg, Benjamin A. Clegg, Francisco R. Ortega

**Affiliations:** 1 Department of Industrial and Operations Engineering, University of Michigan, 1891 IOE Building 1205, Beal Ave, Ann Arbor, MI 48109 USA; 2 Department of Psychology, Colorado State University, 1876 Campus Delivery, Fort Collins, CO 80523‑1876 USA; 3 College of Engineering, University of Notre Dame, 257, Fitzpatrick Hall of Engineering, Notre Dame, IN 46556 USA; 4 Department of Psychology, Montana State University, P.O. Box 173440, Bozeman, MT 59717-3440 USA; 5 Department of Computer Science, Colorado State University, Fort Collins, CO USA

**Keywords:** Attention, Display design, Head-mounted displays, Overlay clutter, Augmented reality, Computational clutter modeling, Human performance, Visual search

## Abstract

This work examines the influence of clutter when presenting information with a head-mounted display (HMD). We compare clutter costs when displays overlay a real-world scene to the costs of visual scanning required when displays are presented separately. Using an HMD in safety–critical environments reduces repetitive visual scanning and head movements that can become effortful with separate displays, such as a tablet. However, a trade-off occurs with overlay displays when low visibility information in the scene is needed or when perceiving text and symbols on the display requires high visual acuity. To examine this scan–clutter tradeoff, participants performed tasks requiring focused attention on either the scene or the display. The HMD either overlaid the critical aspects of the scene or was presented adjacent to the scene. The amount of clutter in both domains was quantified and manipulated. The HMD overlay and adjacent conditions showed similar performance for accuracy, but the overlay condition hindered tasks requiring focused attention on the scene. Perceiving clutter as perceptually closer was attributed to a biological tendency to prioritize information closer to the observer, which disproportionately harmed attention to scene information. Increasing clutter in both domains caused an increasing cost to both speed and accuracy. The results speak favorably to using an HMD, but signal the need to be cautious of the negative effects of clutter in either domain. These results highlight the importance of carefully designing HMDs to minimize clutter, especially when scene information is required.

## Significance

In military operations, fast and accurate decision making is critical, especially when advanced technologies like head-mounted displays present the soldier with increasing amounts of information. The present work investigates the tradeoffs of using a head-mounted display (HMD) to present mission-critical information, emphasizing the performance impacts of increasing amounts of visual clutter. We examined how clutter impacted performance when HMD information was presented as either overlaid onto a simulated real-world scene or side-by-side. While HMDs offer operational benefits, clutter, especially in overlay displays, interferes with the perception of essential scene details. Overall, the results suggest that caution should be taken when considering the negative effects of clutter on both speed and accuracy, especially in safety-critical military contexts.

## Introduction

The head-mounted display (HMD) has proven valuable in many real-world tasks (Dey et al., 2018; Jeffri & Rambli, 2021), in part by rendering display imagery in close proximity with the view of the world beyond. In military contexts, the proximity of information can be crucial for time-critical tasks, such as an attention cue directing a soldier to the location of a landmine. Such presentations can reduce the amount of visual scanning and head movements relative to a separated display like a tablet when information both on the display and in the real-world scene requires attention (Warden et al., 2023a, b). Such visual scanning or *information access effort* between the scene and the display can be substantial when the latter is viewed head down or on a tablet (Poole et al., 2023; Schons & Wickens, 1993; Warden et al., 2023a, b). Numerous examples of HMD benefits have been demonstrated in the literature (see Dey et al., 2018 and Jeffri & Rambli, 2021, for reviews) for domains such as education (Chytas, et al., 2020; Fonseca et al., 2014), manufacturing (Lamberti et al., 2014; Olsson et al., 2009), in-vehicle navigation (Kim & Wohn, 2011; Morrison et al., 2009), aviation (Fadden et al., 2001), maritime (Aylward et al., 2021; van den Oever et al., 2024), and health care (Ameri et al., 2019; Archip et al., 2007). In particular, the *augmented-reality* head-mounted display (AR-HMD) presents virtual and real-world information within the same frame-of-reference and field-of-view (FOV; Milgram & Kishino, 1994), providing an added benefit to human performance beyond reducing the demands of visual scanning relative to a separated display. That is, the AR-HMDs present display images in world-referenced coordinates where the virtual information has a one-to-one correspondence with real-world information, such as a virtual arrow that always points toward a target in the real world as head orientation changes. This feature is advantageous in military operations where spatial awareness and fast and accurate information alignment are mission-critical.

While an HMD provides benefits compared to a more separated display containing the same information, they still come with some drawbacks. One primary cost is the *clutter* imposed when two sources of information are superimposed: specifically, information on the near-domain display and in the far-domain scene beyond the display (Moacdieh & Sarter, 2015, Warden et al., 2022; Warden et al., in press). Clutter has various definitions in the literature (Moacdieh & Sarter, 2015; Kaber et al., 2008; Rosenholtz et al., 2007). Moacdieh & Sarter (2015, p. 5) define clutter as “…the presence of performance and attentional costs that result from the interaction between high data density, poor display organization and an abundance of irrelevant information.” The detrimental effects of clutter are commonly examined in visual search tasks (Wolfe, 2021; Wolfe and Horowitz 2004; see Wickens et al., 2022 for a review), but these effects can also be seen in non-search tasks requiring focused attention. For example, reading a particular word or interpreting a symbol on an HMD or head-up display (HUD) may be hindered by nearby visual information in the scene beyond (Weintraub & Ensing, 1992; Fadden et al., 2000), and detecting objects in the scene can be hindered by overlaying display imagery. The balance between reduced scanning benefits and clutter costs with an HMD defines the scan–clutter trade-off. This study extends the exploration of how spatial alignment of overlaid information and how clutter impacts attentional processing when operators use AR-HMDs, offering novel insights into mechanisms of attention when using AR in real-world settings.

Two primary interests of the current study included (1) determining the overall performance costs of clutter caused by overlaying HMD information on a far-domain scene that was designed to simulate a real-world scene, such as terrain that a soldier may traverse during a mission, and (2) assessing the scan-clutter tradeoff. The costs of clutter can occur in two distinct directions. First, clutter from the far-domain scene, such as the world or an image simulating a real-world scene, can inhibit the search or readout of information on the near-domain display, such as the content presented on a head-up display (HUD) or HMD. In this case, far-domain clutter is created by the complexity of real-world information or a combination of virtual information embedded in the real-world instead of virtual content presented directly on the display. Second, the clutter on the near-domain display can inhibit processing information in the far-domain scene, such as a low-salience target (Fadden et al., 2000). Cost of clutter occurring in these two distinct directions will be referred to as *clutter directionality*. Of equal interest is how clutter directionality effects wered modified by the amount of clutter in each information source—the near-domain display and the far-domain scene.

Examining the impact of varying amounts of clutter requires systematically quantifying clutter in a display. There have been several computational clutter metrics proposed and evaluated in the literature, including a simple count of objects referred to as *numerosity clutter* (Warden et al., 2024; Yeh & Wickens, 2001), a measure of *edge density* which examines edges (i.e., edges of objects like squares or hexagons) and density of objects (i.e., how close objects are relative to each other) in a display (Rosenholtz et al., 2007; Neider & Zelinski, 2011), a measure of *feature congestion* which quantifies basic visual features (e.g., color, luminance, contrast, line orientation) that vary across the display (Rosenholtz et al., 2007; Neider & Zelinski, 2011; Henderson et al. (2009), Treisman & Paterson, 1984), and *overlay clutter* which is a simple dichotomous measure that distinguishes between clutter present when images are superimposed, as in HMDs or HUDs, or clutter absent when equivalent information is displayed on a head-down or otherwise separated display (Warden et al., 2024). Findings from this study will advance our theoretical understanding of costs associated with clutter and of the scan–clutter tradeoff, both of which have not been quantified when information is presented with an AR-HMD.

In addition to examining the scan–clutter tradeoff, our study focused on how increases in clutter, measured by the feature congestion clutter metric (Rosenholtz, 2007), amplified the costs of overlaying information. We also explored whether these increases in clutter affected the near-domain display differently than the far-domain scene. In Warden et al., (2023a, b), we examined both computational and overlay clutter effects for both focused attention and information integration tasks. Building on our previous work that examined these effects using desktop displays (Warden et al., 2023a, b), the current work extended this exploration to the context of an HMD. Using realistic maps and natural scene images, Warden et al. (2024) revealed that overlay displays degraded accuracy for tasks requiring focused attention on either the near-domain display or the far-domain scene compared to adjacent displays. Such a cost was not observed when information integration between the two domains was not required. These findings are particularly relevant for the soldier in the battlefield who must frequently integrate information from multiple sources without losing focus on other important environmental details. Thus, the close display proximity created by overlay is advantageous (Kroft & Wickens, 2002; Warden et al., 2023a, b; Wickens & Carswell, 1995), when two sources of information need to be integrated or compared.

Furthermore, and relevant to the current study, in several measures and different clutter conditions, there was evidence of an asymmetry in clutter directionality between clutter on the display and in the scene when attention needs to be focused on the display or the scene beyond (Warden et al., 2023a, b). That is, clutter on the overlaid display hurt focused attention on the far-domain scene more than clutter in the scene hurt focused attention on the display. This effect was attributed to an evolutionary biological mechanism referred to as *peripersonal space* (Bufacchi & Iannetti, 2018), whereby events closer to the body attract more attention and are given higher priority than more distant events; this is under the assumption that the former is more likely to pose threats to the observer. There are many contexts (Woodward & Ruiz, 2022), such as maritime (van den Oever et al., 2024), surgery (Tu et al., 2024), driving (Arvanitis, et al., 2023), and the military domain, where such an asymmetry could result in prioritizing information on the HMD at the cost of missing other critical information in the environment, which may complicate decision-making. Generalizing this biological depth asymmetry, we assumed that this asymmetry could be attributed to a greater dominance, and greater resistance to clutter, for items on the near-domain display than those in the far-domain scene. In the current study, we provide a unique examination of this asymmetry in the context of a military-based paradigm using an HMD.

The current study replicates some of the same manipulations and methods as Warden et al., (2023a, b; 2024), who simulated an HMD overlay display by imposing overlay between the two sources of information on a flat-panel display. The present study assessed whether the findings of focused attention costs for overlaid displays, and of the clutter asymmetry on the flat screen display replicate and generalize to an HMD with different overlay material and type of task. In the prior study, the scene and display images were superimposed on the same flat-panel display, such that there were no additional depth cues available to help segregate the two sources of information, like stereopsis or relative motion. In contrast, the current study renders the display imagery on an actual HMD, which provides the important and powerful depth cue of binocular disparity to separate the two images in depth. We predict that the addition of this powerful depth cue may make it easier for the viewer to discriminate between the two domains of information, and, therefore, diminish the costs of overlay clutter.

In the current experiment, participants viewed two images, with varying levels of clutter, rendered on an HMD: a schematic map (near-domain display) and a naturalistic scene that simulated the real-world environment (far-domain scene). The two images were presented either directly overlaid or adjacent to each other. Both display configurations mirror tasks that a soldier might perform, where the near-domain display, such as a map, is either presented directly in front of them or off to the side, as in a head-down display (e.g., tablet). This setup required participants to focus on either the near or far-domain, similar to decision-making scenarios encountered by military personnel. We hypothesize that:

- H_1_. Due to overlay clutter, there will be a greater cost to performance on focused attention questions for the overlay display compared to the adjacent display (H_1A_). However, the overall costs of clutter will be smaller than those found in Warden et al. (2024) due to the availability of the optical distance depth cue of binocular disparity in the current study to segregate the two domains of information (H_1B_).
- H_2_: Given that the computational feature congestion clutter model predicts that higher feature variability (i.e., variability in color, orientation, luminance) leads to greater perceptual clutter (Rosenholtz et al., 2007), the model will linearly predict response time and error rate as clutter is varied across different near-domain displays and far-domain scenes. Such linearity of search time as well as subjective ratings with feature density clutter output values has been observed by Nieder & Zelinksi (2011) and Henderson et al (2009).
- H_3_: Given the biological nearness hypothesis and the prior findings of Warden et al., (2023a, b), a clutter directionality asymmetry will be observed, indicating greater costs of near-domain display clutter when focusing attention on the far-domain scene than far-domain scene clutter when focusing attention on the near-domain display.

## Method

### Participants

Fifty-four students enrolled in an introductory psychology course at Colorado State University received course credit for completing the experiment. The average Cohen’s *f* effect size reported in prior research is approximately 0.89 (Warden et al., 2023a, b). Based on this effect size, a post hoc power analysis using a repeated-measures ANOVA and an alpha level of 0.05 indicated that a minimum sample size of 23 participants would be sufficient to achieve 80% power. However, since we were interested in the generalizability and statistical robustness of more complex statistical analyses (i.e., linear mixed models), a larger sample size was deemed necessary. All participants had self-reported normal or corrected-to-normal vision and were screened for colorblindness using an electronic version of Ishihara’s test. No demographic information was collected. This experiment was approved by the university’s Institutional Review Board, and informed consent was obtained.

### Stimuli and apparatus

All stimuli were presented on a HoloLens 2 augmented-reality (AR) HMD. The HoloLens 2 (HL2) is a mixed-reality HMD created by Microsoft Corporation. The lateral and vertical field-of-view (FOV) of the device is 43° and 29°, respectively. The HL2 was part of an incremental examination geared toward bridging the gap between simple 2D displays to more complex real-world scenarios. The experimental environment was developed in the Unity game engine using version 2019.3.1f1 and the Mixed Reality Toolkit (MRTK) version 2.5.1. The Mixed Reality Toolkit (MRTK) is a utility that integrates with the Unity game engine, taking traditional interactive 3D scenes and rendering them in the HL2 while providing useful tools like head/eye tracking, natural interactions with gestures, and stereoscopic rendering.

The near-domain display consisted of a 2D schematic map generated in Adobe Photoshop. The map simulated one that a soldier might use to navigate the terrain while on the battlefield. The map consisted of labels for town names and elevation data and icons representing trees, mountains, towns, rivers, and a representation of the user’s location. We generated the map clutter first by using a count metric for low, medium, and high, where there was an average of 4, 15, and 22, icons, respectively. Then, these map clutter levels were quantified and verified as either low, medium, or high based on the Rosenholtz et al. (2007) feature congestion clutter metric. Collapsing across display and task, the mean feature congestion metric values for low, medium, and high clutter were quantified as 3.08, 3.77, and 4.85, respectively. The feature congestion values quantify the degree to which features in the image create relative visual complexity and crowdedness and, as noted above, have been found to linearly correlate with both search response time, and subjective ratings of clutter. Stimulus materials can be viewed on the OSF page (https://osf.io/zm6gw/).

A total of 18 unique far-domain scenes were generated using Google Street Views from Google Maps. The far-domain scenes simulated a real-world landscape that a soldier might traverse during a battlefield mission. We used real-world images to align with the scenario of a soldier’s task and to ensure that we had some variability in the type of background images. Each raw image was 798 × 798 pixels and consisted of natural landscape imagery extracted from Google Street Views. The contrast and luminance were matched across images. The clutter level of the scenes was quantified as either low, medium, or high based on Rosenholtz’s feature congestion clutter metric. Collapsing across display and task, the mean feature congestion metric values for low, medium, and high scene clutter were quantified as 2.87, 4.47, and 5.73, respectively. It should be noted that all feature congestion values were extracted from the raw images on a desktop computer rather than the images that were projected to the user from the HL2.

In the AR-HMD, the near-domain display was either directly overlaid onto or positioned adjacent to the far-domain scene. Both the near and far-domains were virtual renderings in world-referenced coordinates in the sense that the domains were anchored to an (x, y, z) coordinate in the real world, but neither moved with head orientation. For the overlay condition, the far-domain virtual display was rendered at a slightly further depth plane than near-domain virtual display, creating a sense that the near-domain was in front of the far-domain. The map of the near-domain was positioned centrally and overlaid to enhance the frame-of-reference alignment between the two domains, thereby ensuring spatial orientations were consistent across both domains. Additionally, the map size was determined to ensure the legibility of the information. Display images appeared to participants as 2D virtual planes presented within an augmented-reality space. These virtual display plans were fixed in real-world space as the participant attempted to ascertain information between the images, possibly while moving their head in the process. Only two displays were ever present on a given trial: one of a naturalistic scene (far-domain display) and the other of contextual map information (near-domain display).

**Fig. 1:**
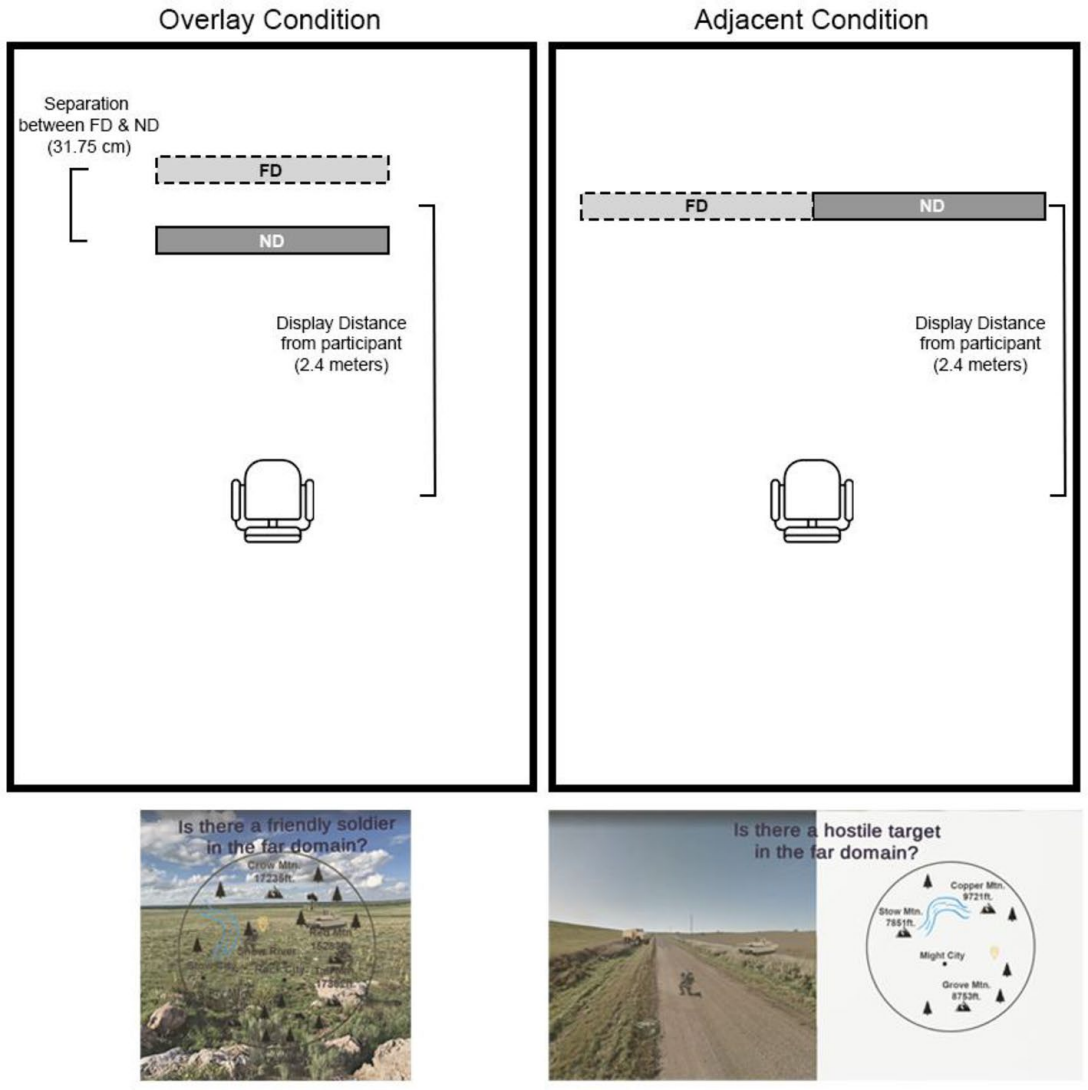
An illustration of the experimental set up for each display condition. Using the Hololens 2, virtual displays were projected to represent the far-domain (FD; light gray rectangle) and near-domain (ND; dark gray rectangle). In the overlay condition (left), the two virtual displays were separated by 31.75 cm and the participant was positioned at the center of the displays located 2.4 m from the midpoint between the two virtual displays. In the adjacent condition, the two virtual displays were side-by-side and the participant was positioned at the center (where the two displays abut) and 2.4 m from the virtual displays. Display conditions were counterbalanced. Participants saw both conditions and were repositioned depending on the display condition they were to complete. The images below each schematic are screen captures from the device and the perspective of the participant

Each virtual display presented in augmented reality was square with approximately 1 × 1-m dimensions. These images were placed in real-world spatial coordinates approximately 2.4 m away from the location of the participant. These dimensions and locations ensured the displays fit well within the FOV of the HL2. Letters within the display were, on average, a minimum of 3.18 cm tall and subtended a visual angle of approximately 0.76 degrees, which is well within the range of readable text (Lee et al., 2017). In the adjacent configuration, the far-domain (real-world) scene was disjoint from the near-domain display. Specifically, the near-domain display was placed adjacently to the right of the far-domain display, such that the edges of both virtual displays were abutted. For the adjacent condition, participants were seated so that the forward gaze was in the middle of both displays, meaning at the point where the two edges of the far and near-domain displays abutted. Thus, there should be no a-priori bias or greater legibility offered to the far-domain display over the near-domain display. The visual angle in this configuration, which includes both displays side-by-side, was 45.2 degrees. The visual angle based on the center of the near and far-domain displays was approximately 23.6 degrees of visual angle. In the overlay configuration, the near-domain display had a transparent background and was overlaid directly on the far-domain display. In this case, the far-domain (naturalistic scene) and the near-domain (map) were separated by 31.75 cm in depth. For the overlay condition, participants were seated so that their forward gaze lined up with the middle of the virtual images. The visual angle of this configuration was 23.6 degrees. While we did not formally assess the legibility of the text for the displays, through pilot testing we ensured that font size did not impose legibility issues for participants with normal or corrected-to-normal vision. Additionally, the HL2 applies anisotropic filtering to images, ensuring that they remain sharp and consistent when viewed at different angles. See Fig. 1 for a schematic representation of the experimental set up for each display condition.

The decision to use virtual 2D imagery to represent real-world scenes was driven by the following considerations. First, the primary goal of the work was to assess the influence of varying levels of clutter in both the near and far-domains, particularly when these domains were overlaid. Using 2D naturalistic images for the far-domain allows us to systematically control and measure the level of clutter in each image. Such control allows us to isolate the effects of clutter. Additionally, using 2D images ensures that all participants were exposed to identical visual conditions without other factors that might impact the findings (e.g., depth perception issues), thereby enhancing the reproducibility and reliability of the findings. Lastly, the images used incorporated all eight static cues for depth—relative size, texture gradient, occlusion, linear perspective, retinal image size, light and shadow, height in the visual field, and aerial perspective. These cues have been shown to effectively convey depth information without the need for binocular vision (Okoshi, 2012; Knill, 2005; Troscianko, et al., 1991, Saxena et al., 2007). Prior work looking at depth cue utilization shows that motion and stereo cues diminish their effectiveness for depth judgments at distances beyond approximately 10 m. The static cues employed in the present experiment for depth judgments on the far-domain scene remain effective across a longer range of natural distances of several hundred meters, typical of the scenes used in this experiment (Cutting & Vishton, 1995). While each of the displays was static images, there was a depth disparity between the near and far-domain in the overlay condition. After assessing how a small amount of depth perception impacts human performance, we can expand our findings to situations where virtual imagery is rendered with stereo and motion depth cues while preserving the 2D overlay information (e.g., map).

### Task

**Fig. 2:**
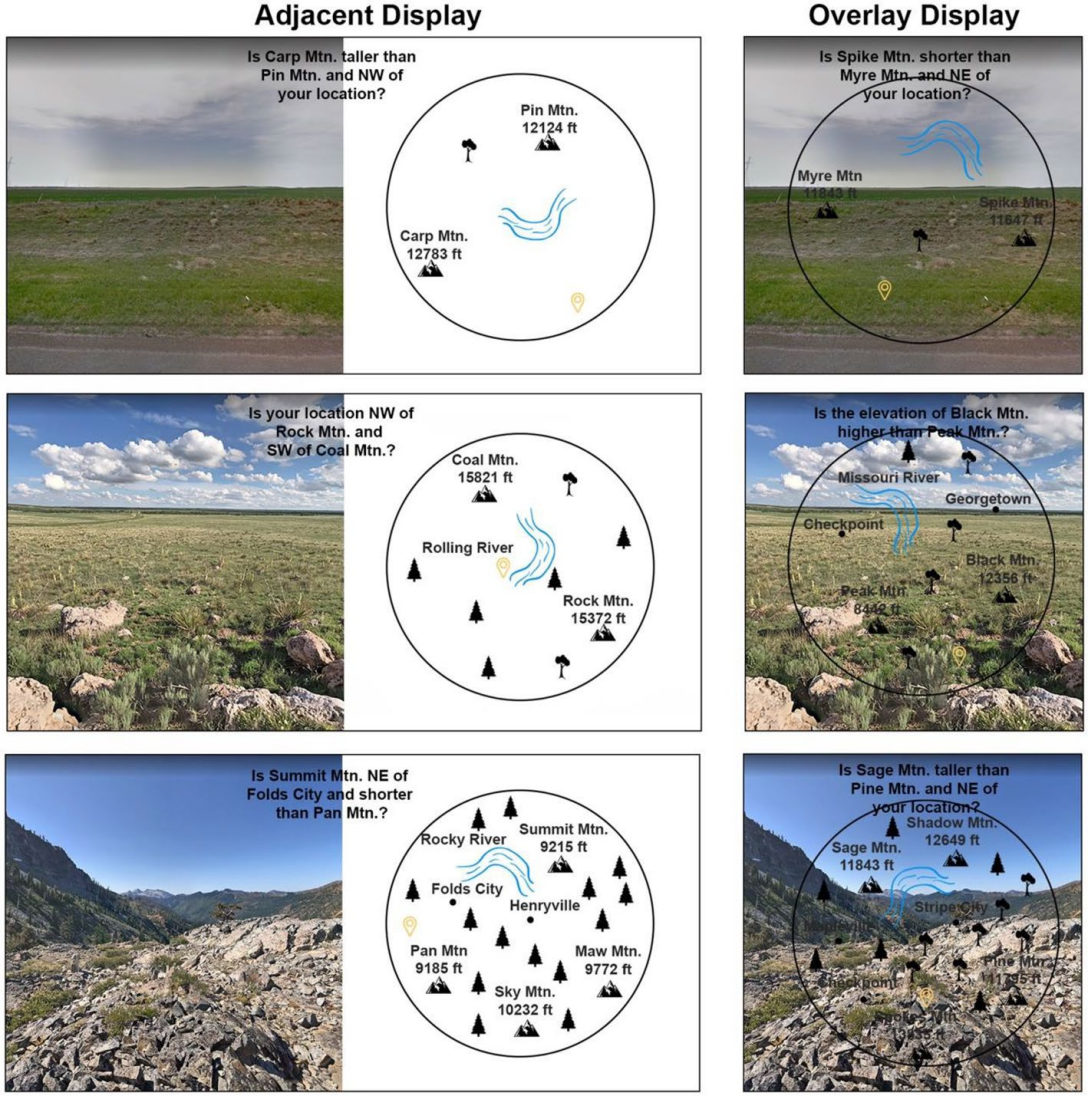
An illustration of each display type with each level of near and far-domain clutter for the near-domain focused attention map task. The top images show low near and far-domain clutter, the middle images show medium near and far-domain clutter, and the bottom images show high near and far-domain clutter. All 9 combinations of near and far-domain clutter were possible

**Fig. 3:**
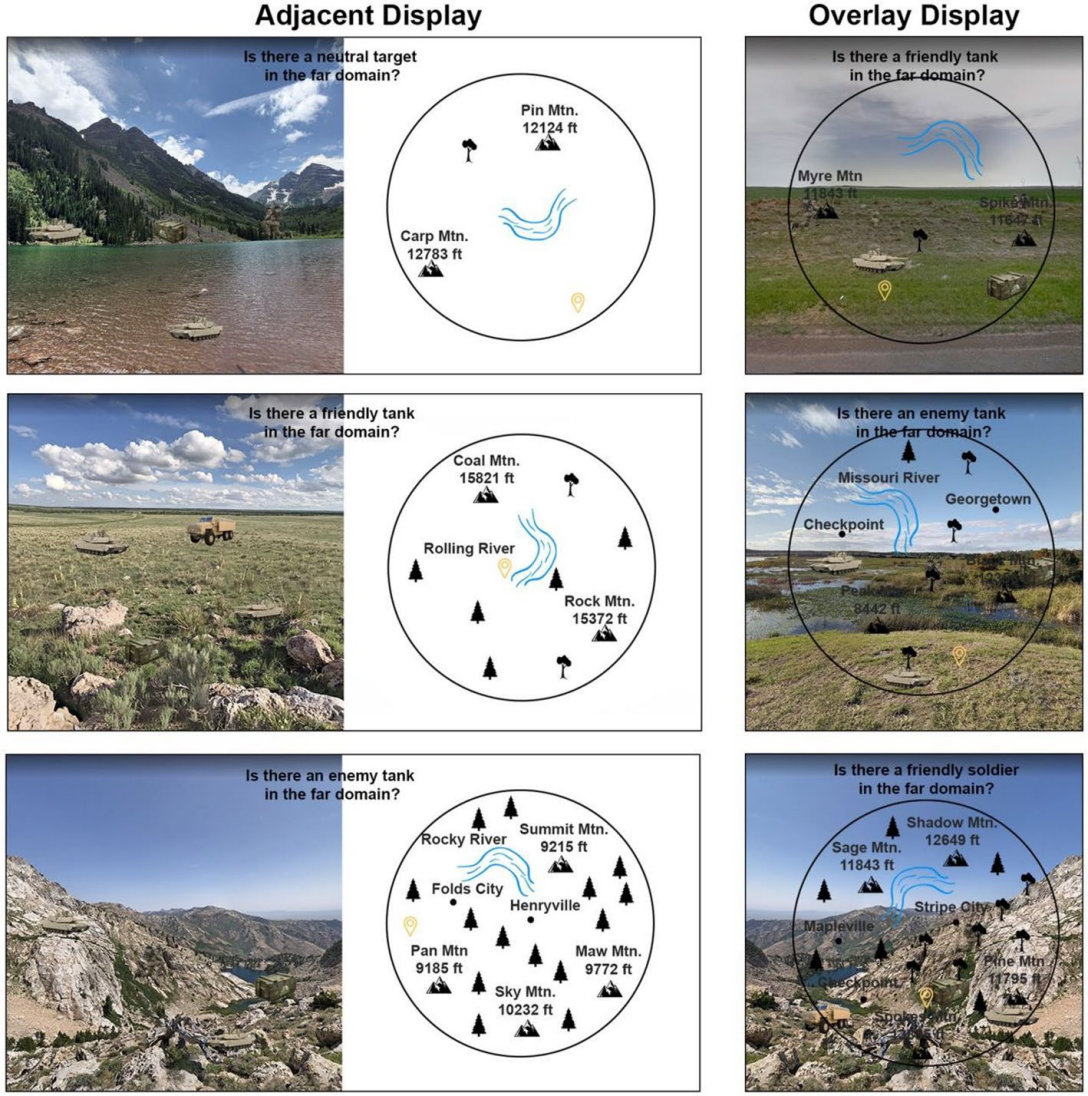
An illustration of each display type with each level of near and far-domain clutter for the far-domain focused attention scene search task. The top images show low near and far-domain clutter, the middle images show medium near and far-domain clutter, and the bottom images show high near and far-domain clutter. In the figure, near and far-domain clutter are always the same (e.g., high/high, medium/medium, low/low), even though in the actual experiment, all 9 combinations of clutter in both domains were used

Participants completed a different type of task for each domain: a scene search task and a display map task, as shown, for the different levels of clutter, examples of which are shown in Figs. 2 and 3. The scene search task in the far-domain scene required identifying if a target was present in the far-domain. Four camouflaged objects were uniformly embedded in the far-domain. The objects were either neutral (friendly soldier, friendly tank, military truck, crate) or hostile (enemy soldier, enemy tank). All friendly targets were oriented leftward and enemy targets were oriented rightward. For example, a friendly soldier faced to the left whereas an enemy soldier faced to the right. All questions for the scene search task pertained to the far-domain image, meaning that focused attention on the far-domain was required. The far-domain scene search task simulates the visual scanning and identification process that soldiers perform on the battlefield, specifically in situations where they must discriminate between friendly or enemy forces. For example, soldiers might need to scan the battlefield for potential threats, such as enemy soldiers or vehicles, which may be camouflaged, before advancing on the terrain.

The map task on the near-domain display required focusing attention on two sources of information presented in the near-domain. For the map task, they had to either determine (1) whether the elevation of one location was greater (i.e., elevation questions) or less than a second location, or (2) whether the user’s position was located in a specific direction relative to another location (i.e., location questions). All questions for the map task required focused attention only on the near-domain display. We were careful to ensure that near-domain display imagery never physically occluded far-domain targets of the scene search task. The map task simulates a soldier’s use of navigational aids, like a map or GPS, to assess terrain features or spatial orientation when traversing the battlefield. For example, soldiers often must assess terrain elevations when they are determining the best vantage point, or they must navigate relative to key landmarks in the world.

Together, these tasks illustrate the complexities behind the tasks that soldiers often conduct during combat where they must switch attention between the far-domain scene (battlefield view) and the near-domain (map) display when processing tactical information from multiple sources.

## Experimental design

The experiment was a 2 (task type: search, map) × 2 (display type: overlay, adjacent) × 3 (clutter: low, medium, high) within-subjects design. Participants completed two blocks, with the order of blocks counterbalanced based on display type (overlay versus adjacent). Each display type block consisted of 72 experimental trials plus six practice trials. The entire experiment had a total of 156 trials. During the practice trials, participants received feedback indicating if they were correct or not. The correct answer for half of the experimental trials was yes, and the other half was no.

Within each display type block, the order of task type (scene search task versus map task) was randomized. The near-domain display map task consisted of a total of 36 trials, 18 of which were location questions and 18 elevation questions. For the location questions, participants were asked something akin to: “*Is your location W of Cedar River and S of Sly Mtn.?*”. For the elevation questions, participants were asked questions like: “*Is Black Mtn at a higher elevation than Kite Mtn?*". Participants were required to respond either yes or no. The clutter level for the near-domain display consisted of 6 trials for each unique level of clutter for both the location and elevation questions. For example, there were six trials of high near-domain clutter for the elevation and six trials for the location questions. Concerning the far-near-domain pairings, the far-domain clutter level varied among these six trials. For each question type, there were two trials for each of the following: (1) high far-domain clutter-high near-domain clutter, (2) medium far-domain clutter-high near-domain clutter, and (3) low far-domain clutter-high near-domain clutter.

**Fig. 4:**
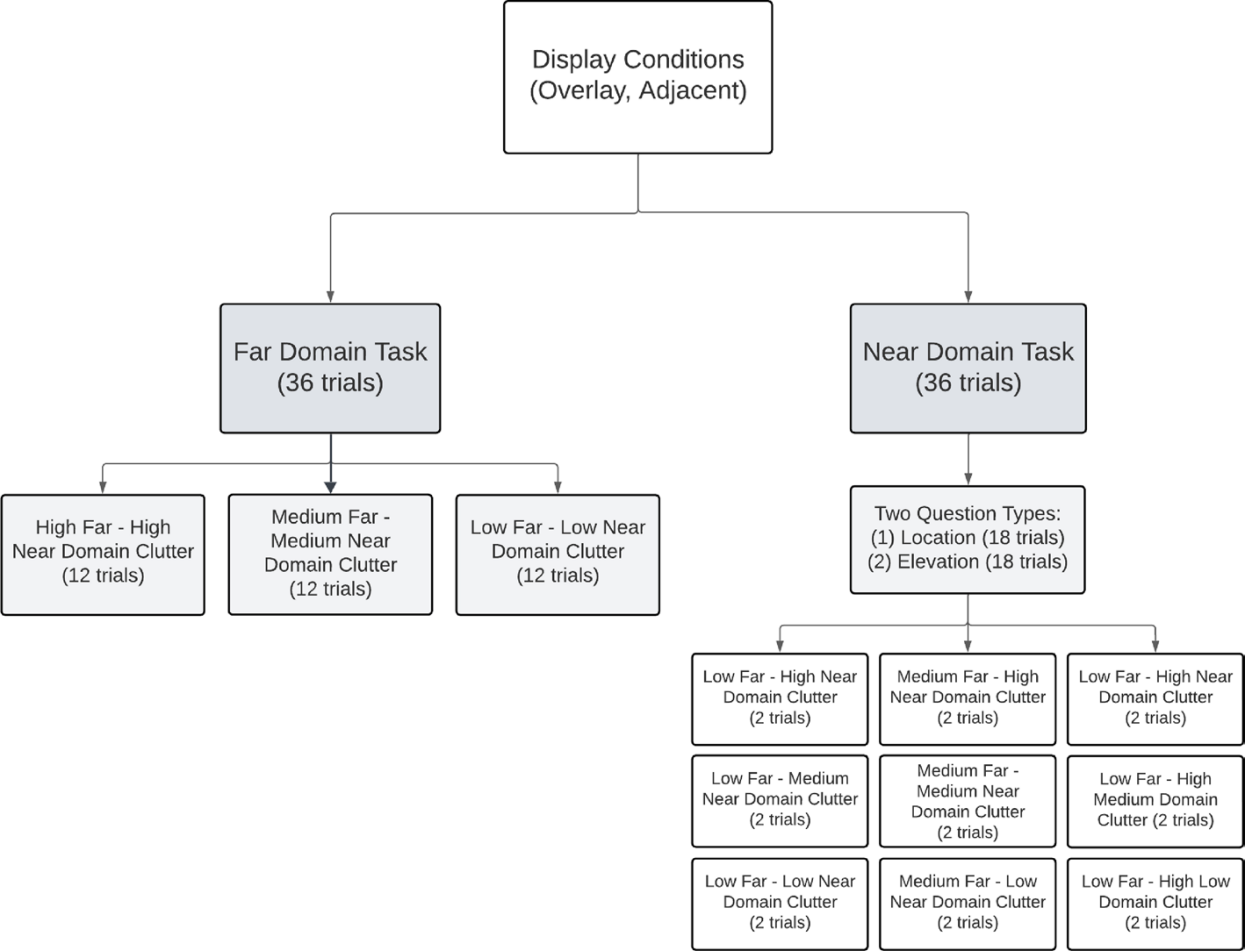
A flow chart showing the Experimental Design. The design was the same for each display condition (overlay, adjacent), which was counterbalanced. All participants completed both display conditions

The far-domain scene search task consisted of 36 trials that required finding a specific target in the scene. For example, participants were asked: “*Is there an enemy tank in the far-domain?*”. The clutter level for the far-domain display consisted of 12 trials for each of the high, medium, and low levels of clutter. It is important to note that for the far-domain task, the far-near-domain clutter pairings always matched (i.e., high far-high near clutter, medium far-medium near clutter, and low far-low near clutter). Figure 4 is a flow chart of the experimental design.

The order of the far-near-domain clutter combinations described above was randomized without replacement for each display block. All participants saw the same stimuli. Performance measures included response time and error/accuracy. Response time was determined from the start of each trial until participants made their yes/no responses using a keyboard. Given that their response was binary, we coded correct responses as correct or incorrect allowing us to compute accuracy (i.e., total correct for each condition) and its inverse, error. The entire experiment lasted approximately 40 min. Participants had the choice to take a break between display conditions. However, no participant chose to take a break. Participants remained engaged during the entire task.

## Procedure

**Fig. 5:**
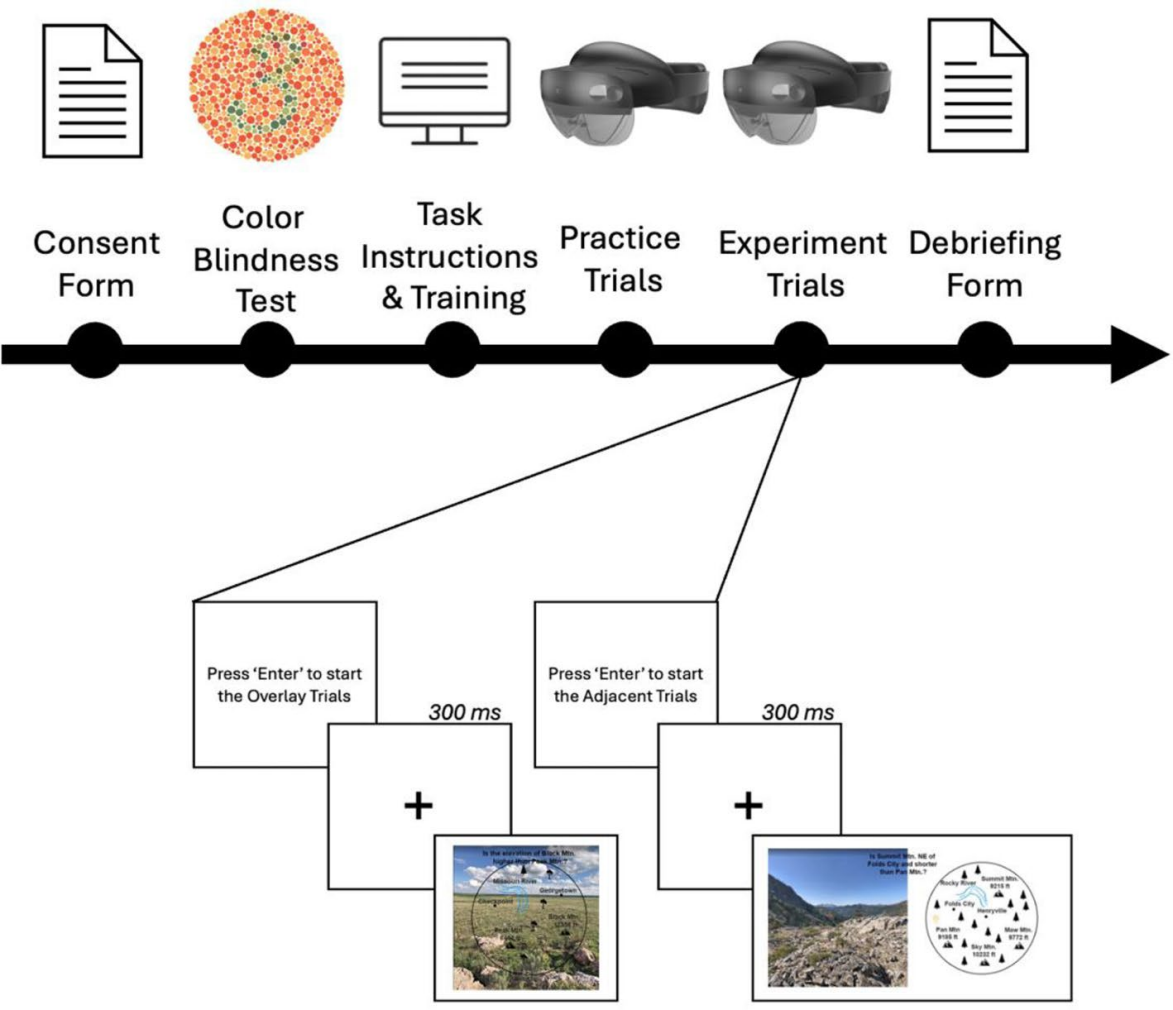
An illustration of the experimental procedure from the perspective of the participant. All participants completed both display conditions, which were counterbalanced by participant

Participants provided their consent to participate after reviewing the consent documentation presented on a desktop computer. All participants completed an electronic colorblindness test which confirmed that no one exhibited signs of red-green colorblindness. Before the experiment, participants completed a training session on reading the maps, understanding the task, and identifying the targets. After the training session, participants put on the AR-HMD to complete the main experiment, which included two rounds of practice trials for each display type before any of the experimental trials. Participants were instructed to answer questions as accurately and rapidly as possible. Participant held a Logitech Bluetooth keyboard in their lap to make their responses. They pressed the ‘caps lock’ key to respond ‘No’ and ‘enter’ key to respond ‘yes.’ See Fig. 5 below for an overview of the experimental trials for each participant.

## Results

Prior to conducting analyses, we used the Grubbs and Rosner’s tests for outliers in addition to examining boxplots. Based on boxplots, five subjects were excluded from the analysis due to two or more instances where unique conditions were 1.5 times the interquartile range (IQR). All accuracy values were below 50% for these outliers, suggesting either task misunderstanding or lack of engagement. All data were analyzed in R Studio (Version 2024.09.0 + 375).

### Information access effort versus overlay clutter

We examined whether performance (response time and error) differed with spatial separation between the two domains of focused attention tasks. Based on the Shapiro Test and Q–Q plots, both response time and percent error violated normality assumptions. For response time, the data were log-transformed to account for this violation. For percent error, transformations did not improve the violation. However, given that the ANOVA is robust to normality violations when sample sizes are moderate to large (Blanca et al., 2017; Schmider et al., 2010), the analysis reported here is still reliable (N = 49).

**Fig. 6:**
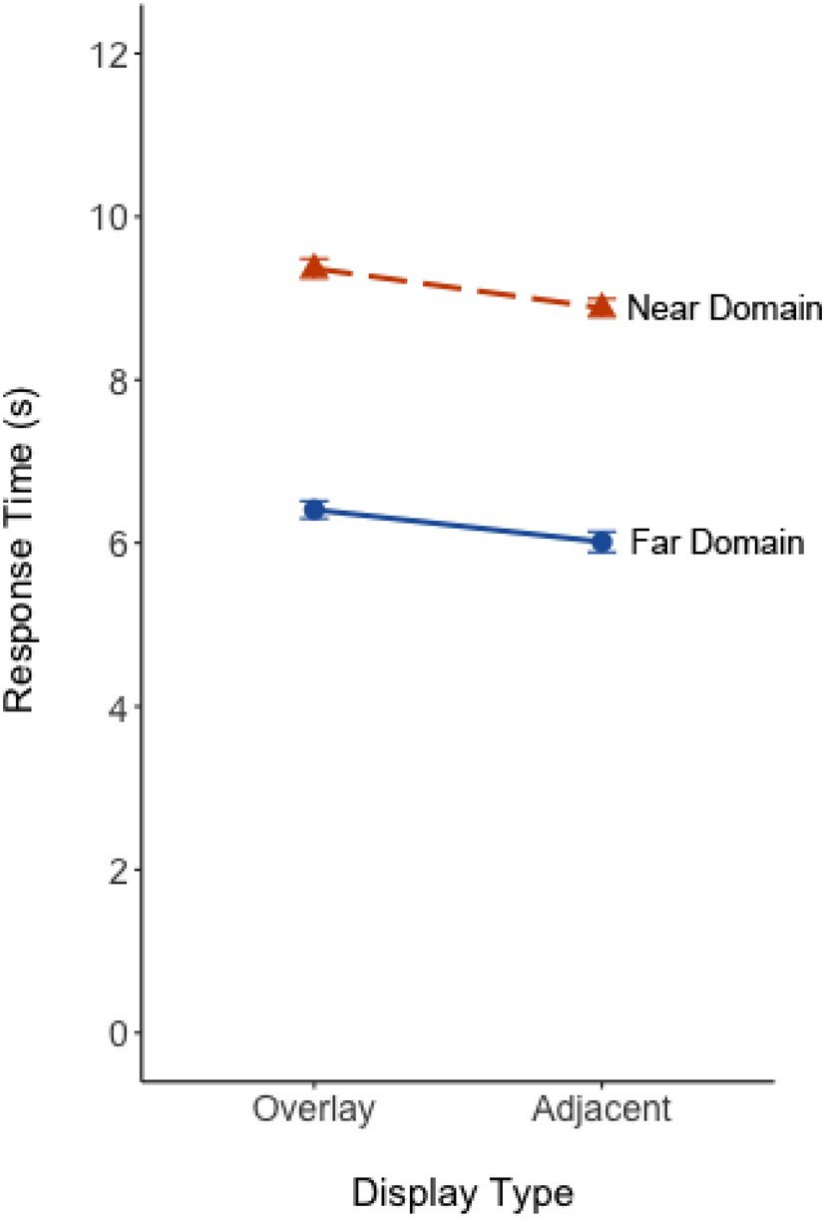
The mean response time plotted as a function of display overlay and focused attention task. Solid blue and dashed orange lines represent focused attention task for the far-domain and near-domain, respectively. Error bars represent one standard error of the mean

Response time data are shown in Fig. 6. Untransformed data are presented for visual clarity, while log-transformed data were used for the ANOVA. There was a statistically significant main effect of task type, *F*(1, 48) = 369.1, *p* < 0.001, η_p_^2^ = 0.88. Participants were 2.9 s (*s*) faster for questions about the far-domain scene (*M* = 6.0* s*) than the near-domain display (*M* = 8.9* s*), where the task required searching for two elements and making a comparison between them. Additionally, there was a statistically significant effect of display type, *F*(1, 48) = 6.49, *p* = 0.014, η_p_^2^ = 0.12. Response time was nearly half a second slower with the overlay display (*M* = 7.90 s) compared to the adjacent display (*M* = 7.45 s). The interaction between task and display was not significant (*p* = 0.34).

**Fig. 7:**
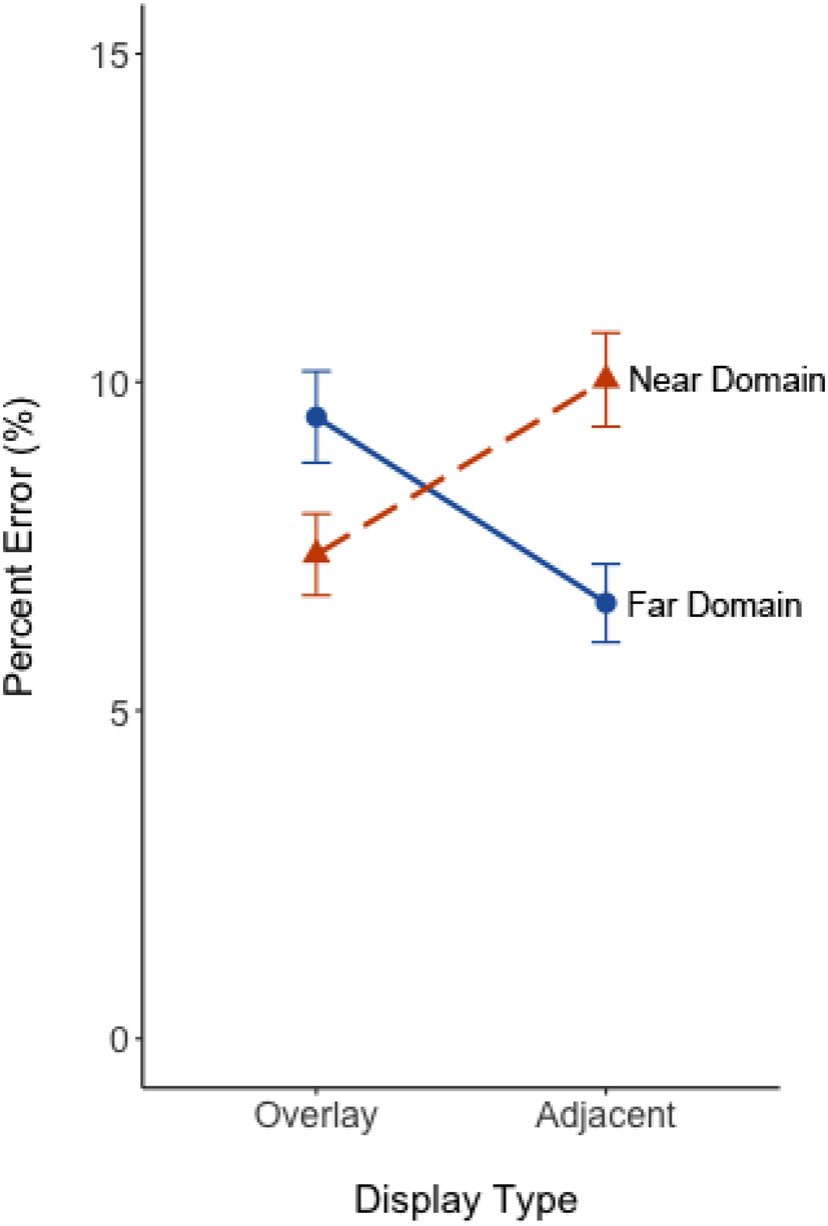
The percent error plotted as a function of display and focused attention task. Solid blue and dashed orange lines represent focused attention task for the far-domain and near-domain, respectively. Error bars represent one standard error of the mean

Percent error data are shown in Fig. 7. The ANOVA revealed no a main effect of task (*p* = 0.40) or display (*p* = 0.91). However, there was a large and significant cross-over interaction between task and display, *F*(1, 48) = 15.65, *p* < 0.001, η_p_^2^ = 0.25. Pairwise t-tests were Bonferroni-corrected. Effect sizes (Cohen’s *d*) are reported for each paired comparison. For the far-domain scene search task, percent error was higher with overlay than adjacent display (*t*(48) = 3.20, *p* = 0.002, *d* = 0.50), suggesting greater performance costs for far-domain information with overlay displays. For the near-domain map task, percent error was lower with overlay displays compared to adjacent displays (*t*(48) = 2.42, *p* = 0.02, *d* = 0.38), suggesting overlay displays were less detrimental for near-domain tasks.

Overall, the focused attention task location (near vs far) impacts response time, while display depth had a differential impact on percent error. Supporting the biological hypothesis (H_3_), we infer that overlay displays showed that near-domain information, being perceptually closer, appeared more accessible and enhanced performance compared to the perceptually distant far-domain scene. Performance worsened for the far-domain task due to the clutter from the near-domain overlay but improved when displays were adjacent and such overlay clutter was removed.

To further examine these effects, we combined the present data with our prior work examining the same effects using a desktop display with similar stimuli and tasks (Warden et al., 2023a, b). We conducted a 2 (experiment) × 2 (display) × 2 (task domain) mixed ANOVA, with display and task as within-subjects factors and experiment as a between-subjects factor for all data.

**Fig. 8:**
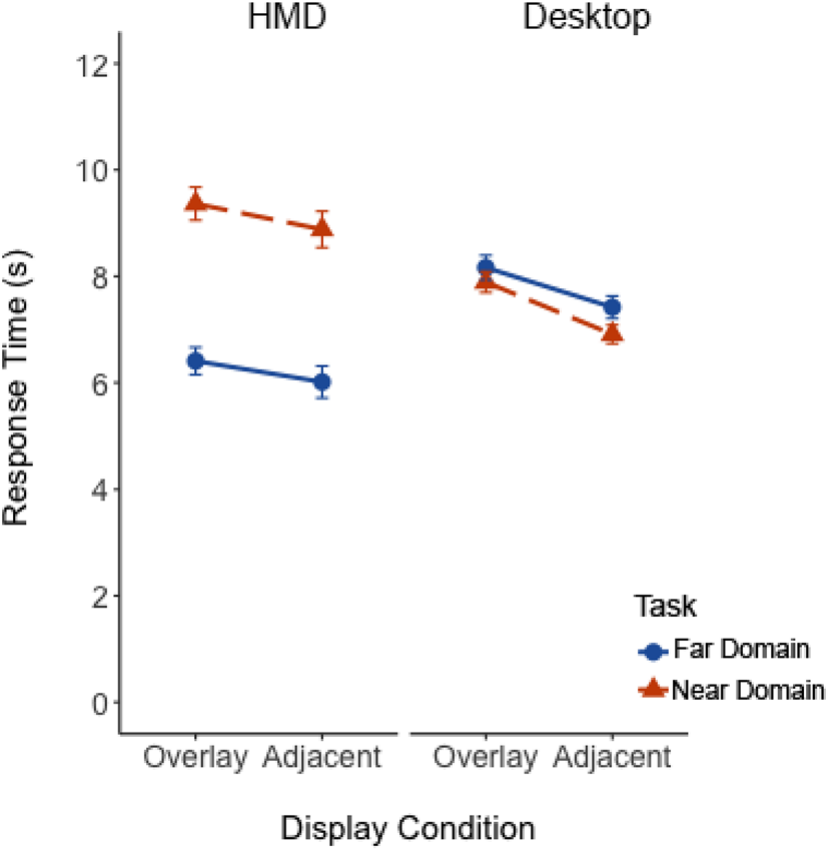
The mean response time plotted as a function of experiment (HMD vs Desktop), display overlay and focused attention task. Solid blue and dashed orange lines represent focused attention task for the far-domain and near-domain, respectively. Error bars represent one standard error of the mean

*Response Time*. The data are shown in Fig. 8. The ANOVA revealed a significant main effect of display on response time, *F*(1, 128) = 19.96, *p* < 0.001, η_p_^2^ = 0.13, suggesting a cost of overlay in both experiments. There was a significant main effect of task on response time, *F*(1,28) = 127.20, *p* < 0.001, η_p_^2^ = 0.18. In both experiments, response times were slower for the near-domain than the far-domain task. Critically, there was a significant interaction between experiment and task, *F*(1, 128) = 218.33, *p* < 0.001, η_p_^2^ = 0.63. In the HMD experiment, near-domain tasks showed increased response times, whereas the response time cost was greater for the far-domain task in the desktop experiment. This discrepancy in the HMD experiment may be due to the additional search requirements imposed by the task.

**Fig. 9:**
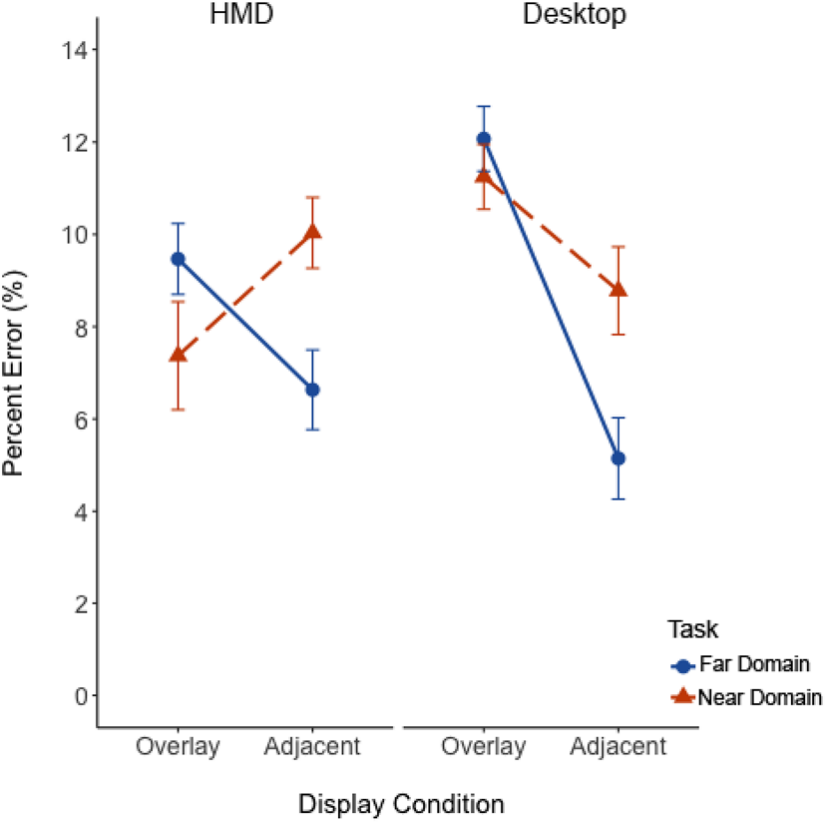
The mean percent error plotted as a function of experiment (HMD vs Desktop), display overlay and focused attention task. Solid blue and dashed orange lines represent focused attention task for the far-domain and near-domain, respectively. Error bars represent one standard error of the mean

*Percent Error*. The mean percent error data are plotted in Fig. 9. The between-experiment ANOVA revealed a significant main effect of display on percent error, *F*(1, 128) = 18.15, *p* < 0.001, η_p_^2^ = 0.12, indicating a higher cost from overlay displays compared to adjacent displays. There was also a significant main effect of task on percent error, *F*(1, 128) = 4.26,* p* < 0.001, η_p_^2^ = 0.03, suggesting more errors in near-domain tasks than far-domain ones. Notably, the interaction between display and experiment was significant, *F*(1, 128) = 16.88, *p* < 0.001, η_p_^2^ = 0.12. When using overlay displays, percent error was higher in the desktop than in the HMD experiment, suggesting the HMD reduced overlay clutter effects. Conversely, adjacent displays caused more errors in the HMD than with the desktop experiment. Lastly, the significant interaction between task and experiment, *F*(1, 128) = 31.38, *p* < 0.001, η_p_^2^ = 0.20, suggests a greater overlay decrement for the near-domain map task on the 2D desktop display but essentially no difference in decrement (Figs. 7 and 9) on the HMD (i.e., the current experiment).

Collectively, overlay displays consistently increased both response time and error rates more than adjacent displays, replicating higher performance costs due to overlay displays. Critically, the additional depth cues provided by an HMD seem to mitigate these costs, particularly to near-domain tasks, unlike desktop displays that lack such depth cues.

## Clutter effects on focused attention

We used linear mixed models and binomial generalized linear mixed models to examine the effects of feature congestion clutter (a continuous variable) on response time and error rate, respectively. Since the assumption of normality was violated, we used log-transformed response time, which reduced the positive skew, improved residual symmetry, and better satisfied the model assumptions. Response time data was used for the graphs for ease of visual interpretation. This analysis was carried out for the overlay display only because we expected the maximum disruptive effects of clutter to emerge in this condition (Warden et al., 2023a, b). Each mixed model includes participant as a random effect (i.e., by-participant random intercept model), which allows us to account for the non-independence that arises in the data when participants complete multiple responses. We also examined the random slopes models, which, in all cases produced the same pattern of results as the intercept only model: estimates, effect directions, and significant values. Therefore, we only report here the intercept only model because these models converged with no singularity issues. We ran the linear mixed models using the lmerTest package (Kuznetsova et al., 2017), which automatically extends the lmer() from lme4 to compute the p-values and degrees of freedom for fixed effects using the Satterthwaite’s approximate to control for Type 1 errors (Singmann & Kellen, 2019). We computed Cohen’s *d* effect sizes by computing the expected mean difference divided by the square root of the expected pooled variance of an individual observation (Westfall et al. 2014).

**Fig. 10:**
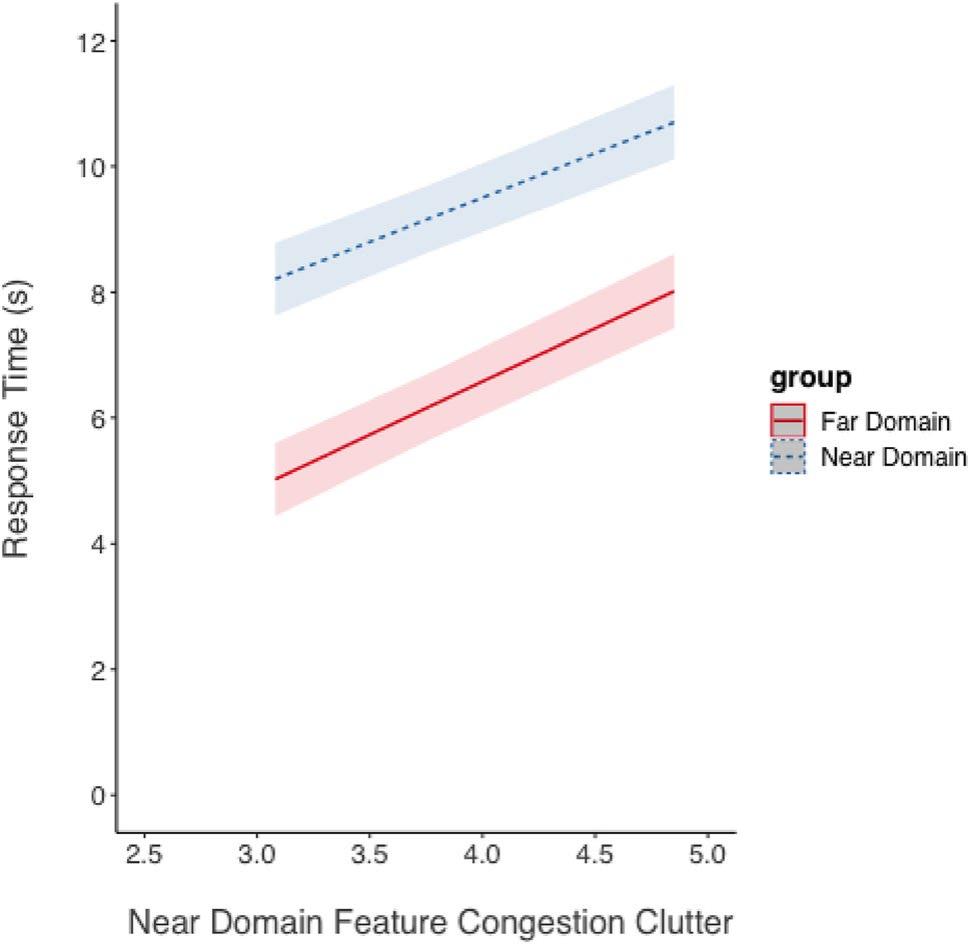
Response time (shown as the marginal mean response time measured in seconds) as a function of near-domain feature congestion clutter and focused attention task domain predicted from the model for the overlay display. Shading represents 95% CIs as calculated from the model

*Response Time*. Figure 10 displays response time data. Each regression line depicts the predicted values for the 3 levels of clutter and values of the predictors in the model. The mean feature congestion value for each level of clutter in the near-domain map image was used: high (M = 5.73), medium (M = 4.47), and low (M = 2.87). The fixed effects were near-domain feature congestion clutter, task domain (far or near-domain), and their interaction. The task domain was submitted as a factor.

The model revealed a large and significant effect of task domain on log-transformed response time, estimate = 0.32, *SE* = 0.04, *t* = 8.28, *p* < 0.001, *d* = 0.1.39. Far-domain (scene search) questions were answered faster than near-domain (map) questions. In addition, there was a significant degrading effect of increasing near-domain feature congestion clutter on response time, estimate = 0.10, *SE* = 0.007, *t* = 14.61, *p* < 0.001,* d* = 0.44. The interaction between near-domain clutter and task was significant, estimate = −0.03, SE = 0.01, *t* = −3.37, *p* < 0.001,* d* = 0.14. Increasing clutter in the near-domain led to longer response times in both conditions, but this increase was more pronounced for the far-domain tasks, suggesting that participants were more sensitive to clutter in the near-domain when searching the far-domain. While this interaction is significant with log-transformed response time data, it is important to note that the interaction was not significant with the untransformed response time data (estimate = −0.28 s, *SE* = 0.20, *t* = −1.42, *p* = 0.16, *d* = 0.06.), although the pattern of the interaction is trending in the same direction for both models.

**Fig. 11:**
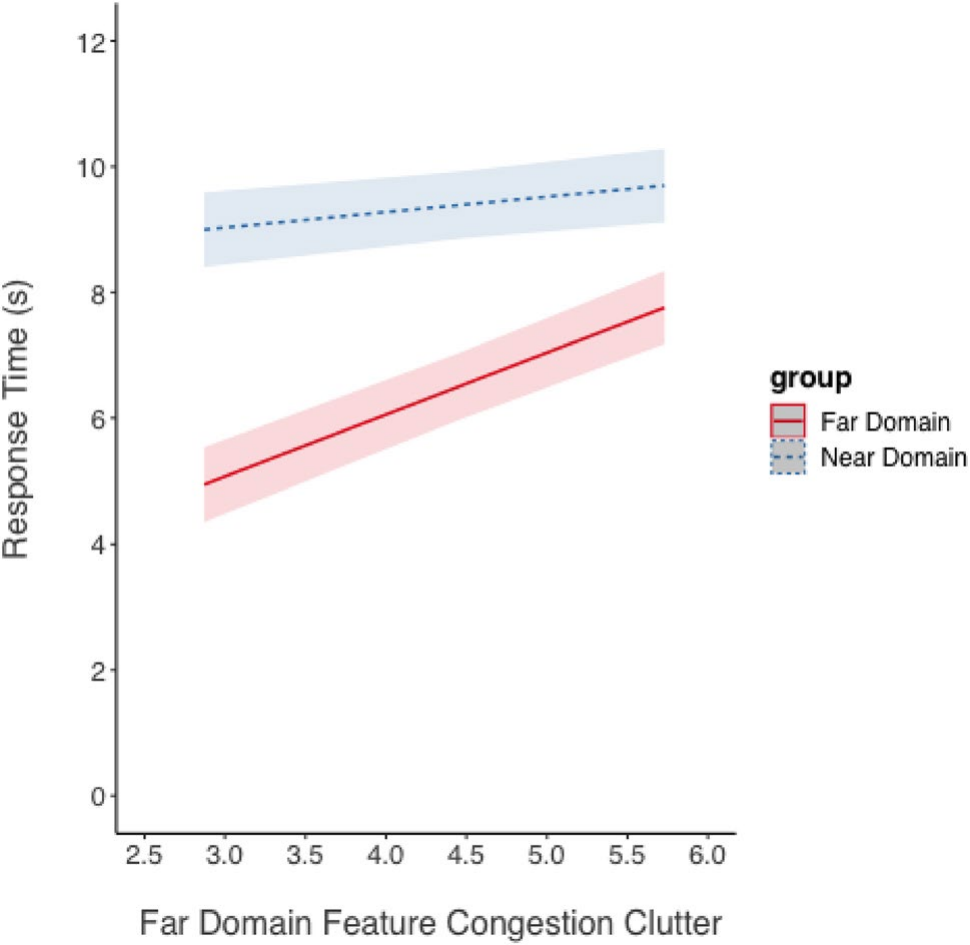
Response time (shown as the marginal mean response time measured in seconds) as a function of far-domain feature congestion clutter and focused attention task domain predicted from the model for the overlay display. Shading represents 95% CIs as calculated from the model

The same analysis was conducted for far-domain feature congestion clutter using the mean far-domain feature congestion value for each level of clutter in the far-domain scene image (see Fig. 11). The model revealed a large and significant effect of task domain on log-transformed response time, estimate = 0.38, *SE* = 0.03, *t* = 13.70, *p* < 0.001, *d* = 1.63. In addition, there was a significant degrading effect of increasing far-domain feature congestion clutter on response time, estimate = 0.06, *SE* = 0.004, *t* = 13.55, *p* < 0.001, *d* = 0.25. The results are best interpreted in the context of the significant interaction, estimate = −0.04, *SE* = 0.006, *t* = −7.03, *p* < 0.001, *d* = 0.19. The degrading effect of increasing far-domain clutter was greater for tasks relevant to the far-domain than tasks relevant to the near-domain.

**Fig. 12:**
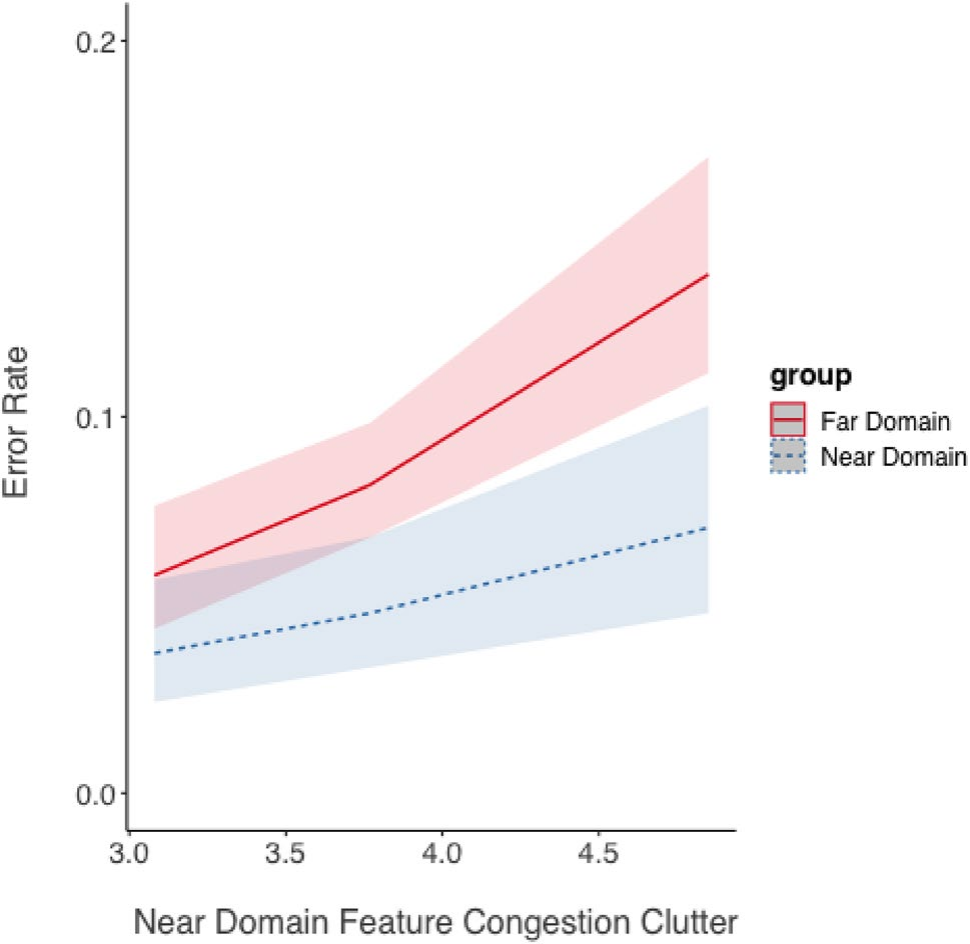
Error rate (shown as the marginal mean) as a function of near-domain feature congestion clutter and focused attention task domain predicted from the model for the overlay display. Shading represents 95% CIs as calculated from the model

*Error Rate*. The effects of near and far-domain feature congestion clutter on error rate was analyzed essentially the same was as described above, given the binary coding of accuracy. As shown in Fig. 12, there was a significant degrading effect of increasing near clutter on error rate for both task domains, estimate = 0.54, *SE* = 0.11, z = 4.81, *p* < 0.001. Neither the effect of task domain (near or far) nor the interaction between task domain and clutter level was statistically significant (both *p* > 0.10).

**Fig. 13:**
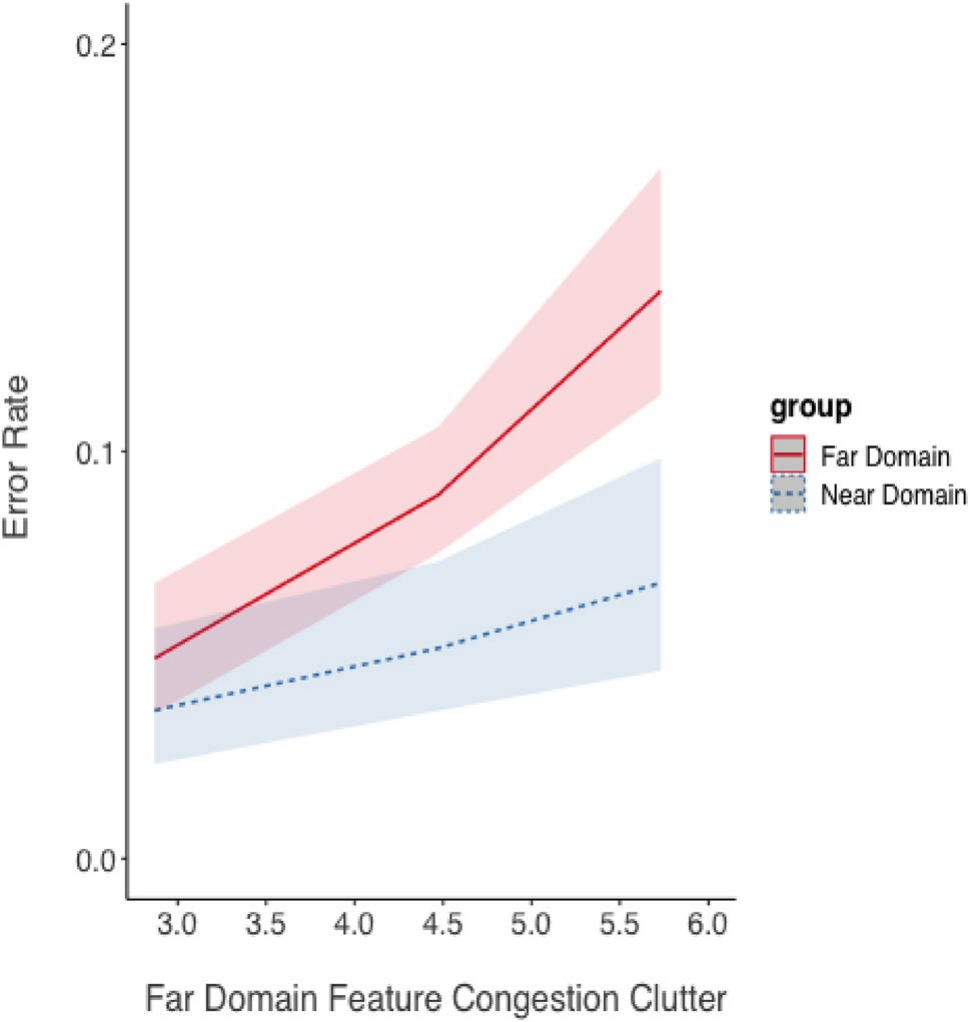
Error rate (shown as the marginal mean) as a function of far-domain feature congestion clutter and focused attention task domain predicted from the model for the overlay display. Shading represents 95% CIs as calculated from the model

The second analysis revealed a significant effect of far-domain feature congestion clutter on error rate, estimate = 0.40, *SE* = 0.08, *z* = 5.26, *p* < 0.001. Error rate increased as far-domain clutter increased regardless of the task (Fig. 9). Neither the effect of task nor the interaction was significant (*p* > 0.10) (Fig. 13).

## Discussion

The current experiment examined performance costs associated with imposing an overlay display and increasing amounts of display clutter presented in an AR-HMD for military related tasks requiring focused attention, and the extent to which those costs are mitigated by separating information. A primary purpose was to examine the extent of the scan–clutter tradeoff when evaluated in an HMD. Prior research in this area has been conducted with a flat panel display depicting images of the display and the scene beyond (Warden et al., 2024, 2023a, b) comparing images directly overlaid or those presented adjacently. Related research has also examined this trade-off for overlaid and adjacent images not designed for an HMD (Kroft & Wickens, 2002; Wickens & Ward, 2017). Importantly, these prior studies were carried out with the two images depicted at a single depth plane, and they have all revealed that, for focused attention tasks, the costs of overlay were substantial.

In contrast, the current study used an AR-HMD to simulate a real-world scene image, where an overlaid screen-fixed display of information was presented at a closer depth plane than the real-world scene image. By doing this, we could assess how information presented with an AR-HMD modulates attention and, therefore, performance. We hypothesized (H_1_) a cost of clutter (H_1A_) but expected this cost would be greatly reduced compared to that observed in prior studies (H_1B_) due to the availability of a depth cue, specifically stereopsis, which assists in differentiating the near- and far-domain images. Prior work observed a 0.5 s overlay cost to response time (Warden et al., 2024), which was attributed directly to the costs of clutter when perceptually segregating the two domains. The second part of this hypothesis was strongly supported by the mixed ANOVA comparing the current results with those from Warden et al. (2024): specifically, the current experiment eliminated any speed or accuracy cost of overlay clutter.

We also hypothesized (H_2_) a linear relationship between the performance decrement and the amount of clutter as quantified by Rosenholtz’s feature congestion metric. Linearity was observed in prior work (Neider & Zelinski, 2011; Henderson et al., 2009) and in our prior research (Warden et al., 2024). But, as noted, these studies had been carried out on 2D flat panel displays, and only Warden et al. (2024) simulated HMD imagery. In the current work, H_2_ was strongly supported as shown by the highly significant linear correlation indicating that response time and error rate increased as clutter increased in either domain. Overall, both clutter models show that increasing near-domain clutter imposed costs to performance (both response time and accuracy). The increasing clutter effect here replicates the previous study which revealed a greater performance cost to processing *far-domain information* as near-domain clutter increased (Warden et al., 2023a, b) compared to processing near-domain information as far-domain clutter increased. In the present study, clutter did significantly interact with the task domain: as near-domain (overlay) clutter increased, the visual processing load became more demanding when focusing attention on the far-domain scene than the near-domain map. Despite the perceived difference in depth between the near-domain display and far-domain scene, this spatial separation did not fully prevent interference. Instead, the findings suggest the overlay clutter in the near-domain captured attention and caused visual competition, thereby impairing performance for the far-domain tasks. However, it should be noted that the model only reached statistical significance using log-transformed response time data. While the model with untransformed response time data was trending in the same direction, the interaction was not significant, suggesting some uncertainty. Thus, although spatial separation may provide participants with a visual depth cue that information was separated, it may not fully eliminate interference under conditions of increased near-domain clutter. This finding contributes to the understanding of how spatial properties and information density interact with information presented in AR to influence performance.

Our third hypothesis (H_3_) predicted an asymmetry in clutter directionality due to the biological mechanism referred to as peripersonal space (Bufacchi & Iannetti, 2018), where near-domain display clutter would impose greater costs when focusing attention on the far-domain than when focusing attention on the near-domain display. This hypothesis was based on trends evident in the data from Warden et al. (2024). This hypothesis was partially supported, as indicated by the significant cross-over interaction found in percent error (Fig. 7) and the interaction between task and FC clutter for response time (Fig. 10), where focused attention on the far-domain was impaired more for the overlay display but focused attention on the near-domain was not. These results support the idea that there may be a biological bias to allocate more attention to information perceived closer to the viewer than information perceived further away, as conveyed by the lower percent error on near-domain questions. That this was not detected across both display conditions may suggest that overlay technology could disproportionately impact cognitive processing due to its immersive nature, which may exaggerate the perceived closeness of visual information.

A second way to examine the biological hypothesis (H_3_) was to look for an asymmetry in clutter costs as the feature congestion metric of clutter increased. This comparison is best illustrated in Fig. 10. These response time data support the biological hypothesis (H_3_), based on the significant interaction between far-domain and near-domain clutter. However, the asymmetry is not supported for accuracy. Instead, the results show that the effects of clutter in each domain were independent for both variables: only additivity was observed, indicating the combined effect of clutter was the sum of the effects of clutter in each domain separately.

Both response time and accuracy increased as feature congestion near-domain clutter increased, supporting H_3_, and suggesting that increasing overlay display clutter hurts speed more for far-domain information but hurst accuracy equivalently for both near and far-domain information.

While the magnitude of the clutter manipulation is equivalent between the near and far-domain (compare Figs. 10 and 11), it is possible that some other clutter metric than feature congestion may have shown a greater near-domain clutter effect, and therefore the amount of clutter, rather than optical distance, might have caused the asymmetry of influence between the near and far-domain. Also, it may be that the feature congestion metric, while quantitatively informative, may not qualitatively capture the type of clutter that impacts cognitive processes differently in near versus far-domain spaces. Including an additional metric of clutter, such as one that assesses cognitive load, may provide more a comprehensive understanding of how clutter impacts attention in different spatial contexts. Future work should seek to assess these effects with additional metrics of clutter and by comparing more of the nuances of the overlay (including difference distances of the overlay relative to the user) versus separate displays.

Our findings highlight the importance of designing AR systems that can effectively manage information presented on an AR-HMD, especially when information is overlaid. These findings also have implications for basic research concerning visual perception and attention allocation of information presented in AR. By understanding how overlaid information impacts the operator’s performance in processing underlying scene information, AR developers can improve how information is layered in HMDs. A key finding shows that separating information across different depth planes can mitigate accuracy costs typically associated with overlaid displays, but costs to response time still occur. This suggests that military AR systems may be enhanced for some tasks by strategically layering critical information at various depths to aid in differentiating important elements in the user’s field-of-view. Additionally, the linear relationship between clutter and performance highlights the importance of emphasizing clutter management in AR interfaces. For military uses, where information must be processed rapidly, displays must be designed to minimize clutter to enhance operational efficiency and decision making. This may be accomplished by adaptively modifying the transparency or color of the overlay display based on its depth relative to the viewer and the scene beyond, ensuring important information remains prominent. Each of these issues becomes more complex in a dynamic, real-world application. While our findings do not fully capture the impact of depth changes, this highlights a gap in the research that must be explored to improve the design of AR systems in dynamic environments.

## Limitations and future directions

One limitation of the research to generalize to naturalistic use of the HMD is our use of virtual content to simulate the scene, even as we did present the HMD information at a different optical distance. A second limitation is that we did not incorporate additional depth cues to differentiate the two domains, particularly relative motion or motion parallax through head rotation, which would be present in a true HMD examining a distant scene. The presence of motion parallax might further enable discrimination and amplify still further, the benefits of overlay. Future research will examine these issues. A third limitation is that we did not account for added aspects of possible confusion between the two domains, related to contrast and color similarity. From our prior research (Warden et al., 2023a, b), we know that these effects are important when displays are overlaid. A fourth limitation is that we did not exploit the 3D rendering capabilities of the device which can allow for examining information in all directions around the user. However, there are significant technical complexities and computational demands associated with 3D rendering that could introduce variability in the visual information. We acknowledge the limitations of not fully using the devices 3D capabilities and emphasize this work sets the stage for future work examine the effects with real-world scenes. While convergence and accommodation may contribute to performance costs observed in the current work, they do not fully explain the performance costs. First, prior work (Warden et al., 2023a, b) has validated costs of clutter with 2D displays. The current work shows the effects of clutter were additive, suggesting costs are due to cognitive and perceptual demands when processing cluttered information instead of strictly due to convergence and accommodation.

While our experiment does provide insights for the presentation of static imagery, we acknowledge that additional challenges arise in dynamic real-world environments where depth and focus are in constant flux*.* And at distances less than about 10 m, such differences are signaled, in part, by binocular disparity (Cutting & Vishton, 1995). Future work that leverages the eye tracking capabilities of the device is needed to explore the impact of dynamic scenes on performance for tasks requiring various attentional demands. Doing so may provide further support for the biological nearness argument if the findings show that gaze involuntarily shifts focal depths between the near- and far-domain information. This future work would help refine AR design guidelines for managing rapid changes in visual focus, which are crucial for military operations.

We also acknowledge that the HL2 is limited as to what it can currently offer a soldier in the field. In its current form, the device is not capable to be used in real-world scenes demonstrated in the far-domain. Due to current limitations of the device, it is critical to understand how it can be used to help future development, and this is one way to understand its use in the field and what effects may generalize to its use in the field, especially given that such a device is of great utility in training soldiers. In assessing the influence of the amount of displayed information on performance, future work should quantitatively examine what range of visual angles subtended by the information can be used effectively without performance costs. Such work will shed light on what size and amount of information remains effective.

## Conclusions

The current work advances understanding concerning how AR-HMDs can be optimized for military tasks by examining the impacts of display clutter and spatial separation on performance. Prior work has demonstrated that the HMD, with its overlay of a scene beyond the display, is generally superior to a separated display configuration whether that is a head-down tablet, or a display viewed to the side of the forward view of the scene (Warden et al., 2023a, 2024). This superiority is particularly prominent when information from the scene must be integrated with information on the display, a task type not examined here. But the HMD can also be superior for focused attention tasks as well, given the depth cue of stereopsis provided when separating the two domains. In the current study, we show how much the HMD advantage is offset as clutter increases in both the scene and on the display. The observed reduction in overlay costs suggests that AR-HMDs, which present near-domain information that overlays a real-world scene, offer an effective way to manage clutter, especially compared to traditional flat panel displays. Additionally, these findings show that quantitative models of clutter are particularly valuable for predicting this increasing cost of clutter. Overall, these findings have practical implications for how AR-HMDs can enhance battlefield performance, especially in cluttered environments where soldiers need to respond quickly and accurately.

## Key points

- We compared the HMD advantage of reducing scanning via overlaying images to its disadvantages, such as the cost of clutter. Our findings show that the display image might obscure details of the scene or the scene might disrupt the perception of the displayed information.
- Using Rosenholtz computational clutter model, we show that one can quantify the amount of clutter, and therefore harm, that overlay displays impose on the real-world scene and the display.
- The negative effects of clutter were less severe when using am HMD to present images at different depths. This contrasts with results from work looking at the effects of overlay clutter on a traditional 2D desktop display where images show the same depth.
- The present work suggests the use of clutter models can be employed when establishing guidelines for maximum clutter presented on HMDs. Additionally, experimental stimulations should strive to employ depth cues, such as the separation of information planes, for evaluation.

## Declarations

### Open practice statement

All data, analysis scripts, and stimuli can be found at https://osf.io/zm6gw/. For any additional information, please contact the corresponding author at: acwarden@umich.edu. None of the experiments in this manuscript were preregistered.
